# Nasal Catarrh

**Published:** 1897-02

**Authors:** 


### NASAL CATARRH.

The demands upon an all around practitioner of medicine, like the large body of the profession, who, from choice or necessity, are doing a country or village practice, often tax his resources to the utmost. He is expected to be up with the latest teachings on every subject, and to be ready, without preparation, to treat anything, from a sore finger to a case of typhoid fever; and in surgery, from opening an abscess, to a laparotomy for a ruptured tubal pregnancy; and—as a matter of fact—most of them are—ready, if not prepared,—to do it. Unlike his more favored brother of the city, he cannot turn the case over to a specialist, nor can he have the benefit of consultation, without great expense to his patron. Hence, having to rely upon himself, it is necessary that he shall read, and keep informed, at least, upon the practice in all its branches. To him—most frequently, the specialist is out of the question.

And, as a matter of fact, the sending of patients to specialists is very much overdone. It is, of course, encouraged by the specialists; but there are few, comparatively, of one's general run of patrons who can afford to go to a city for treatment, or who are willing to do so. We do not decry the specialists by any means; they are a necessity, and often, a harbor of safe and successful refuge, alike to the doctor and his patient. The idea we wish to convey is, that it has become so much a fashion, as it were, to refer certain cases to the specialist, that many abuses of the privilege doubtless occur; there are many cases so turned off that could and should be successfully treated by the general practitioner, if he just thought so; and as is most frequently the case in sections remote from cities where he has no alternative, and must think so, he takes hold accordingly. Hence the recorded experience of others, in any given disease, constitutes, for the isolated practitioner, the most valuable, helpful and welcome literature.

These remarks apply more forcibly, perhaps, to what is called by common consent, "nasal catarrh," than to any other disease or condition. The idea has been disseminated, and is generally held, that nasal catarrh is something terrible, and can only be successfuly handled by the city specialist, who must of necessity, it is supposed, have an expensive and impressive array of instruments and appliances; that the disease requires special skill and a special technique of a very difficult kind.

This is partly true; and doubtless there are cases far advanced and neglected that are beyond the resources of the general practitioner; cases, for instance, of atrophic rhinitis, in which already the deeper structures are involved; but everything that is called "nasal catarrh" is not of this formidable character. Indeed, I may say, the majority of cases are amenable to successful treatment by the general physician, and to turn them over to a specialist is to perpetrate a wrong, both upon himself and his patron. There is no more nasal catarrh now, than there was formerly, though one hears much more of it. It is because the nose and throat have been chosen as an exclusive field by certain physicians who have especially prepared themselves for this branch, and provided the necessary apparatus and appliances for its practice, and the idea has been encouraged that only those physicians can cure the disease.

Nasal catarrh is a term that embraces quite a wide pathological range. Most frequently, when first observed, it is only a chronic "bad cold," a sub-acute or chronic rhinitis, accompanied by the distinctive features of an inflamed mucous membrane,—secretion or hypersecretion of mucus, with more or less swelling. The three stages into which, clinically, the disease is divided: "chronic rhinitis," "hypertrophic rhinitis," and "atrophic rhinitis" are arbitrary, and denote only three degrees of the inflammatory process through which any inflamed mucous membrane must go if let alone; it is the beginning, the progress and termination;—congestion, — hypertrophy, — and ulceration (atrophy, or sloughing).

To treat the disease at any stage before the last mentioned condition is reached, requires neither great skill nor special knowledge; nor is it necessary to have a special equipment. Every physician should have in his office a nasal speculum, a fountain syringe and a head mirror. He may or may not be possessor of a laryngoscope, for the diagnosis is often the easiest. There is presented to the doctor a local disease, an inflamed mucous membrane, more or less extensive. It will be found covered with mucous secretion. There is always some swelling, and the symptoms will be correspondingly severe. There is a sense of discomfort; of fullness in the frontal sinus; headache, difficulty of breathing, and, in more advanced stage—a rise of temperature and general feeling of malaise; mouth breathing becomes a habit, and the voice is affected.

In the graver cases, where necrosis of the nasal bones has set in,—in addition to the local symptoms, which are intensified, the secretion becomes offensive;—there may be those of toxæmia from purulent absorption. The latter state will most frequently require the services of the specialist.

But, in the general run of cases, before structural changes have occurred, the indications for treatment are clear and simple.

First, the nasal passages and the throat should be cleansed thoroughly of all mucus; and, often the mucus will be found dry and adherent, and not so easily dislodged, and may require to be removed with forceps. But ordinarily the object can be easily accomplished by means of a tepid solution of salt, administered by means of the fountain syringe. If the mouth be kept open; and the stream introduced up one nostril it will be discharged through the opposite one, without strangling or inconveniencing the patient, and the force of the stream is usually sufficient for a thorough cleansing.

After this preparatory step the indications for treating a simple inflamed mucous membrane suggest themselves: slight astringent and detergent lotions. If to these properties in the wash are added those of an antiseptic and deodorant, so much the better. This treatment should be applied twice daily; morning and evening.

For this treatment Bermingham's* nasal douche is especially and admirably adapted. It is necessary that the head should be thrown well back and the nozzle introduced, when the flow of the fluid may be regulated by the finger on the vent. The mouth should be opened, to prevent unpleasant consequences.

A preparation embodying all the requisites of a lotion admirably adapted to meet the above-mentioned indications is Glyco-Thymoline (Kress). It is a pleasant aromatic mixture—possessing astringent—alterative, detergent, antiseptic and deodorant properties, and can be depended upon to meet the indications for treatment in a very large number of cases of catarrh in either of the earlier stages; and as an auxiliary to other measures will be found especially beneficial in the graver cases.

---

*A small boat-shaped glass vessel of 2 oz. capacity; price to physicians, about 15 cents.

The proper strength for ordinary use is about 25 per cent; that is, to three teaspoonfuls of tepid water add one teaspoonful of Glyco-Thymoline. It is recommended to order the preparation in original packages—15 oz.—to prevent substitution. The name of the preparation indicates its composition.

A young lady, aged 22, of a delicate appearance, presented herself for treatment, believing she was in the earlier stages of consumption. She had catarrh of the nose and pharynx, in the hypertrophic stage. The secretion was abundant, ropy and tough. The swelling obstructed the posterior nares so as to alter the voice, affect the hearing and compel her to breathe through the mouth. She suffered constantly from a dull frontal pain,—neuralgia—she called it. She was feeble and feverish, and had no appetite; languid, and "tired all the time," she said. She suffered moreover with insomnia. After the very first cleansing of the passages as above described, she experienced marked relief; and three weeks' treatment with Glyco-Thymoline alone, in the strength indicated, one part to four of tepid water, effected to all appearances a perfect cure, and I had the satisfaction to see the roses return to her cheeks and lips—the elasticity to her step and the lovelight to her eye. She regained her appetite and had begun to take on flesh when I saw her last.

This is but one case, it is true; but the result of the treatment was so immediate and striking that it gave me confidence both in my ability to treat such cases without any great parade of equipment, and in the preparation Glyco-Thymoline, which is an elegant pharmaceutical, pleasant to the patient, and efficient in its action, as a cleansing antiseptic and deodorant, admirably suited to the purpose for which it is here used and recommended.

